# Evidence of Anti-tumoral Efficacy in an Immune Competent Setting with an iRGD-Modified Hyaluronidase-Armed Oncolytic Adenovirus

**DOI:** 10.1016/j.omto.2018.01.003

**Published:** 2018-01-31

**Authors:** Ahmed Abdullah Al-Zaher, Rafael Moreno, Carlos Alberto Fajardo, Marcel Arias-Badia, Martí Farrera, Jana de Sostoa, Luis Alfonso Rojas, Ramon Alemany

**Affiliations:** 1 ProCure Program, IDIBELL-Institut Català d’Oncologia, L’Hospitalet de Llobregat, Barcelona, Spain

**Keywords:** oncolytic adenovirus, iRGD tumor-penetrating peptide, immune response

## Abstract

To enhance adenovirus-mediated oncolysis, different approaches that tackle the selectivity, tumor penetration, and spreading potential of oncolytic adenoviruses have been reported. We have previously demonstrated that insertion of the internalizing Arginine-Glycine-Aspartic (iRGD) tumor-penetrating peptide at the C terminus of the fiber or transgenic expression of a secreted hyaluronidase can improve virus tumor targeting and spreading. Here we report a new oncolytic adenovirus ICOVIR17K-iRGD in which both modifications have been incorporated. In xenografted A549 tumors in nude mice, ICOVIR17K-iRGD shows higher efficacy than the non-iRGD counterpart. To gain insights into the role of the immune system in oncolysis, we have studied ICOVIR17K-iRGD in the tumor isograft mouse model CMT64.6, partially permissive to human adenovirus 5 replication, in immunodeficient or immunocompetent mice. Whereas no efficacy was observed in the immunodeficient setting due to insufficient viral replication, partial efficacy and a polymorphonuclear and CD8+ T cell infiltrate were observed in the immunocompetent mice. The results indicate that the elicitation of a virus-induced anti-tumoral immune response is responsible for the observed partial anti-tumoral effect.

## Introduction

Growing clinical evidence indicates that adenoviruses are promising agents for cancer treatment. Adenovirus type 5 (Ad5), the serotype most widely used as an oncolytic agent, has been genetically modified with tumor-targeting ligands, promoters, and mutations that confer selectivity and with sequences encoding for genes (also known as armed oncolytic viruses) that increase their anti-tumor activity.^1^ Our group has described several of these modifications by means of a virus platform that tackles the constitutively active retinoblastoma pathway in tumor cells.^2^ The ICOVIR15K adenovirus harbors three modifications: a deletion of the pRB-binding site of E1A (delta24 mutation) and the insertion of E2F sites in the E1a promoter for tumor-selective replication, and the replacement of the KKTK motif of the fiber shaft with RGDK for tumor targeting. Further modifications have been put forward by introducing different transgenes connected to the major late promoter by means of a splicing acceptor.^2^ As an example, ICOVIR17K (also known as VCN-01) is an oncolytic adenovirus derived from ICOVIR15K armed with the secreted hyaluronidase gene to enhance virus spreading within the tumor mass. Hyaluronidase is expressed using a splicing acceptor that links its expression to the major late promoter. This virus has shown favorable safety and efficacy profiles in several preclinical models and is being evaluated in clinical trials.^3^, ^4^ In another modification aimed at improving tumor targeting and penetration, we have incorporated the internalizing Arginine-Glycine-Aspartic (iRGD) peptide into the C terminus of the ICOVIR15K fiber as a fiber-iRGD fusion protein, resulting in ICOVIR15K-iRGD. The iRGD peptide with the sequence CRGDKGPDC is a cyclic peptide identified in an *in vivo* phage-display screening for peptides able to extravasate tumor blood vessels in a metastatic tumor model.^5^ It has been shown that the iRGD peptide targets the tumor vasculature via RGD motif-mediated binding to avβ3- and avβ5-integrins expressed on tumor endothelial cells. After binding to these integrins, a proteolysis mediated by an unknown protease results in exposure of a cryptic arginine-rich peptide motif (R/KXXR/K) that when present as a C terminus (C-end rule) is able to bind to neurophillin-1 (NRP1). NRP1 is overexpressed on angiogenic blood vessels and tumor cells, and iRGD binding to NRP1 activates an endocytic pathway that leads to tumor penetration of iRGD, as well as drugs coadministered or conjugated with iRGD. When the iRGD sequence is fused to the C terminus of a protein, it is able to promote its tumor penetration. We have reported that inserting the iRGD sequence at the C terminus of the fiber as a fusion fiber-iRGD protein in the oncolytic adenovirus ICOVIR15K enhances virus targeting and penetration, resulting in a remarkable anti-cancer effect.^6^

Here we report the fusion of iRGD at the C terminus of the fiber of a hyaluronidase-armed oncolytic adenovirus, generating ICOVIR17K-iRGD. With these two modifications, we aim to simultaneously improve tumor targeting with iRGD and intratumoral dissemination with hyaluronidase. We have studied the efficacy conferred by these modifications in immunodeficient, as well as immunocompetent, mouse models.

Because fully immunocompetent models require engraftment of isogenic murine tumor cells and adenoviruses are species specific, immunotherapy studies with human oncolytic adenoviruses in immunocompetent mice have been difficult to perform.^7^ To overcome this limitation, we have described a murine cell line clone, CMT64.6, derived from the CMT64 murine lung adenocarcinoma cell line of C57BL/6 mice, which is semi-permissive to human adenovirus replication.^8^ Here we explore the anti-tumor activity of the new generated virus ICOVIR17K-iRGD in this model. In addition, given that immune responses to tumor antigens secondary to oncolysis can be affected by the route of adenovirus administration, we have compared the systemic intravenous (i.v.) and intratumoral (i.t.) administration routes, the two most common routes used in clinical trials,^9^ for their ability to induce anti-tumoral immunity.

## Results

### iRGD Enhances the Infectivity and Cytotoxicity of RGDK-Modified Adenoviruses

**Figure 1:**
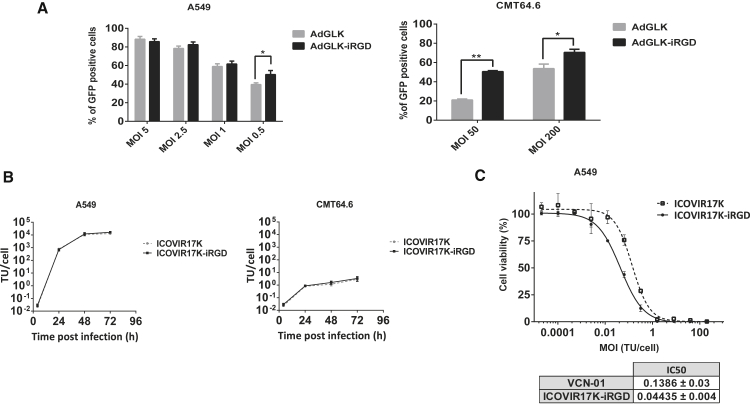
*In Vitro* Characterization of iRGD-Modified Adenoviruses (A) The cell lines A549 and CMT64.6 were infected with the indicated GFP-expressing vectors at different MOIs, GFP was measured after 24 hr by flow cytometry, and the percentage of GFP-positive cells is plotted for each vector. The vectors share the same expression cassette containing GFP and luciferase transgenes. Mean ± SD error bars are plotted. Significant difference between the vectors (*p < 0.05, **p ≤ 0.01) was calculated by two-tailed unpaired Student’s t test. (B) Viral production of ICOVIR17K-iRGD. A549 and CMT64.6 cells were infected with ICOVIR17K or ICOVIR17K-iRGD at the indicated time points, and cell extracts were harvested and titrated. Three replicates were quantified for each cell line. Mean ± SD of triplicates is shown. (C) Comparative cytotoxicity *in vitro* of ICOVIR17K and ICOVIR17K-iRGD. A549 cells were infected with the indicated viruses at doses starting from 200 TU/cell. IC_50_ values (TU per cell required to cause a reduction of 50% in cell culture viability) at day 6 after infection are shown. Four replicates were quantified. Mean ± SD of triplicates is shown.

To check the effect of iRGD modification on virus infectivity in CMT64.6, a subclone of CMT64 selected for higher adenovirus production levels,^8^ we performed an infectivity assay with luciferase and GFP-expressing non-replicative vectors AdGLK and AdGLK-iRGD. Analysis by flow cytometry 24 hr post-infection showed that insertion of iRGD increased viral infectivity (Figure 1A). However, CMT64.6 cells were infected less efficiently than the human reference A549 lung adenocarcinoma cell line, which is widely used to characterize adenoviruses;^10^ for example, at an MOI of 5, 90% of A549 cells were infected, whereas at an MOI of 50, only 50% of CMT64.6 cells were infected.

Having observed the advantage of iRGD in the GFP-luciferase vector setting, we then inserted iRGD at the C terminus of the hyaluronidase-armed ICOVIR17K virus, using the same methods as described for ICOVIR15K-iRGD.^6^ A virus production assay was performed at different time points in the A549 and CMT64.6 cell lines (Figure 1B). Whereas A549 produced approximately 10^4^ transducing units (TUs)/cell, CMT64.6 produced 5 TU/cell, confirming previous results.^8^ To determine whether ICOVIR17K retains its oncolytic properties after iRGD modification, a dose-response cytotoxicity curve was performed in A549 cells (Figure 1C). The calculated half-maximal inhibitory concentration (IC_50_) value of ICOVIR17K-iRGD was 3.1-fold lower than that of its parental counterpart. This lower IC_50_ value indicates that iRGD does not reduce the cytotoxicity potential of ICOVIR17K.

### iRGD Improves the Oncolytic Efficacy of ICOVIR17K

**Figure 2:**
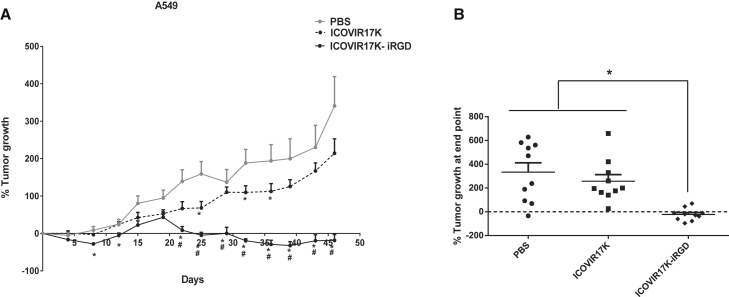
iRGD Improves the Oncolytic Efficacy of ICOVIR17K/VCN-01 in Nude Mice Nude mice bearing A549 lung carcinoma subcutaneous tumors were systemically treated with a single dose of PBS or 4 × 10^10^/mouse of ICOVIR17K or ICOVIR17K-iRGD. (A) Percentage of tumor growth at the indicated days. Mean ± SEM is plotted (n = 10). Significance (*p < 0.05) by two-tailed unpaired Student’s t test compared with the PBS group (#p < 0.05) by two-tailed unpaired Student’s t test compared with the ICOVIR17K group. (B) Percentage of tumor growth distribution at the end of the experiment (day 46).

We next investigated the effect of iRGD on the anti-tumor efficacy of ICOVIR17K. Nude mice bearing human lung adenocarcinoma A549 subcutaneous xenograft tumors were treated systemically via tail vein with PBS or 4 × 10^10^ virus particles (vps)/mouse of ICOVIR17K or ICOVIR17K-iRGD (Figure 2A), and tumor volume was measured twice a week during the experiment. Statistical analysis of tumor growth rate showed significantly improved efficacy of ICOVIR17K-iRGD compared to PBS-treated mice from day 22 post-treatment until the end of the experiment (46 days) and compared to ICOVIR17K from day 29 until the end of the experiment. ICOVIR17K-iRGD significantly reduced tumor growth at the end of the experiment compared to both control groups: 3.6-fold versus ICOVIR17K (p = 0.001) and 5-fold versus PBS (p = 0.0008) (Figure 2B). Although ICOVIR17K-iRGD did not lead to complete regression among the 10 tumors measured, 3 tumors partially regressed and 5 tumors remained stable. Given the strong anti-tumor efficacy of ICOVIR17K-iRGD in nude mice with fully permissive human tumors, we decided to test its efficacy in the most challenging setting of immunocompetent mice.

### Anti-tumor Efficacy of ICOVIR17K-iRGD in Immunocompetent Mice

**Figure 3:**
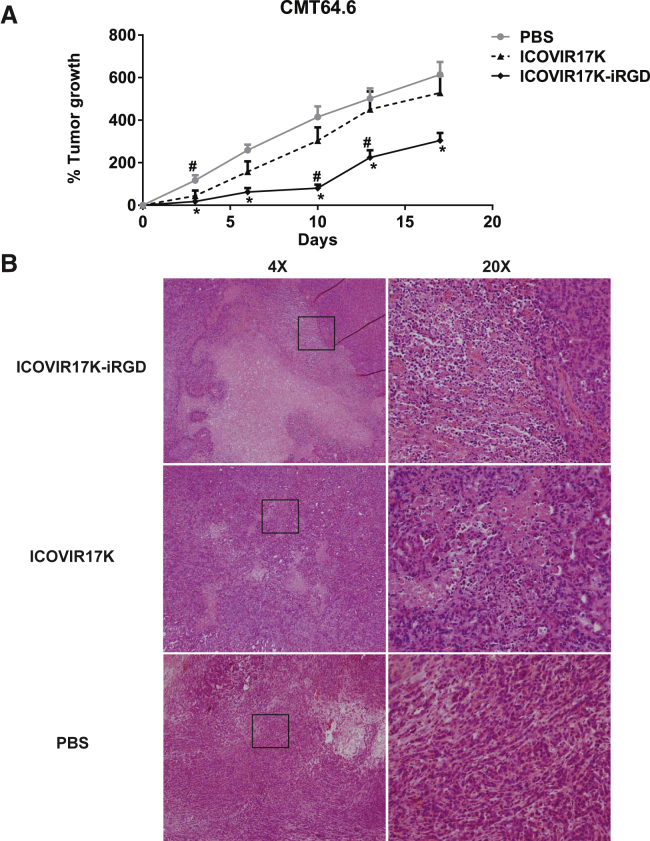
Anti-tumor Efficacy Comparison of ICOVIR17K and ICOVIR17K-iRGD in Immunocompetent Mice C57BL/6 mice bearing CMT64.6 murine lung carcinoma subcutaneous tumors were systemically treated with a single dose of PBS or 3 × 10^10^/mouse of ICOVIR17K-iRGD or ICOVIR17K. (A) The percentage of tumor growth (mean ± SEM) at the indicated days is plotted (n = 12). Significance (*p < 0.05) against PBS, and significance (#p < 0.05) against ICOVIR17K by two-tailed unpaired Student’s t test. (B) H&E staining for paraffin tumor sections. Nuclei stained in blue correspond to granulocytes.

Oncolytic virotherapy with human adenoviruses in immunocompetent mice is limited by poor human adenovirus replication in mouse cells and fast immune-mediated virus clearance. To palliate the first problem caused by species-specificity of adenoviruses, we used a murine cell line, CMT64.6, which is a subclone of CMT64 described by our group as semi-permissive to adenovirus replication (100-fold lower virus production than the reference human A549 cell line).^8^ Mice were treated systemically via tail vein with PBS or 3 × 10^10^ vp/mouse of ICOVIR17K-iRGD or ICOVIR17K (Figure 3A).

The ICOVIR17K-iRGD-treated group showed a slower tumor growth rate compared to the ICOVIR17K and PBS groups, with significant differences starting at day 3 and lasting until the end of the experiment (day 17), when the tumor size was 1.7-fold lower compared to ICOVIR17K (p = 0.001) and 2.2-fold lower compared to PBS (p = 0.001).

At the end of the experiment, tumor sections were stained to detect virus (E1A protein). At this late stage (day 17), virus was not detected in tumors, presumably due to immune clearance. However, H&E staining showed dense infiltration of polymorphonuclear leukocytes (granulocytes) in the tumors of mice treated with ICOVIR17K-iRGD and very weak infiltration in ICOVIR17K-treated tumors (Figure 3B). This experiment confirmed the partial efficacy of ICOVIR17K-iRGD, in contrast to the lack of efficacy of the parental ICOVIR17K, in this mouse model (Figure 3A).

**Figure 4:**
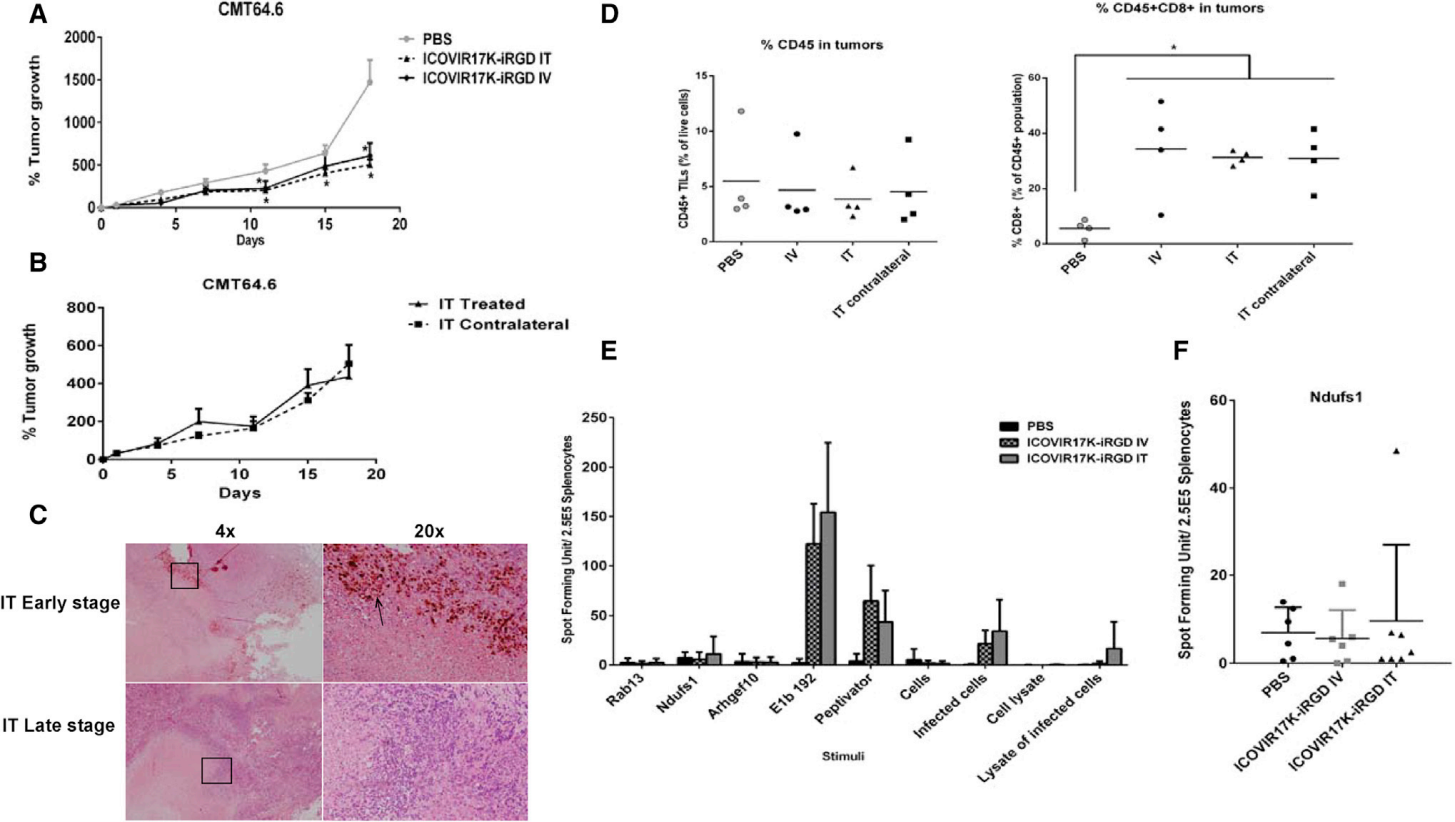
Anti-tumor Efficacy of Systemic or i.t. Administration of ICOVIR17K-iRGD in Immunocompetent Mice C57BL/6 mice bearing CMT64.6 murine lung carcinoma subcutaneous tumors were systemically treated with a single dose of PBS or 3 × 10^10^/mouse of ICOVIR17K-iRGD. (A) The percentage of tumor growth (mean ± SEM) at the indicated days is plotted (n = 11–12). Significance (*p < 0.05) by two-tailed unpaired Student’s t test compared with the PBS group. (B) Comparison of injected and non-injected contralateral tumors of the i.t. mice group. Percentage of tumor growth (mean ± SEM) at the indicated days is plotted (n = 5–6). (C) Immunohistochemical staining with E1A antibody of representative i.t. injected tumors at day 3 or at day 18 post-injection. (D) Levels of CD45 and CD45/CD8 cells in tumors from mice treated i.v. or i.t. (injected and non-injected contralateral tumors). Tumors were mechanically disaggregated and incubated for 30 min at 37°C in RPMI supplemented with collagenase. Single-cell suspensions were prepared, and then cells were stained for viability with Live/Dead fixable stain followed by flow cytometry analysis. (E) Evaluation of immune responses by IFN-γ-ELISpot assay. Splenocytes were harvested and used for analysis of T cell responses 18 days post-treatment (late stage). Three minimal epitopes from CMT64.6 sequencing were synthesized as minimal peptides to detect neoepitope-specific responses. E1b192 peptide and PepTivator (a pool of hexon peptides) were used to detect the immune response against adenovirus. The response to infected and non-infected cells (as whole cells or lysates) was also used to measure the response to the virus (infected conditions) relative to the response to the tumor (non-infected conditions). The graph shows representative results from 18 mice (n = 6 per group). (F) The response against the epitope Ndufs1 by each tested mouse.

To assess the effect of the route of delivery and to gain insights into the mechanisms of action, we compared i.v. and i.t. administration routes for anti-tumor efficacy. CMT64.6 subcutaneous tumors were grown in both flanks of C57BL/6 mice, and mice were treated with PBS systemically, ICOVIR17K-iRGD systemically, or ICOVIR17K-iRGD i.t. Virus-treated groups were given a dose of 3 × 10^10^ vp/mouse. To explore a contralateral effect potentially associated with an immune response, i.t. administration was performed in only one tumor of each mouse. At an early stage of the experiment (day 3 post-treatment), one mouse from each group was sacrificed and tumors were kept in paraffin for immunohistochemistry. The remaining tumors were collected at the end of the experiment (day 18; late stage). Tumors were split for immunohistochemistry and immune response studies; spleens were also collected for immune response studies. Virus-treated groups showed the same tumor control rate regardless of the administration route, which was significant compared to PBS (Figure 4A). In the i.t.-treated group, injected and contralateral (non-injected) tumors responded equally (Figure 4B). We were able to detect virus in tumors at day 3 post-treatment in the i.t. injected tumors, but not in non-injected contralateral tumors or in tumors from mice treated by the systemic route. At day 18, virus could not be detected in any tumor (Figure 4C). All tumors from virus-treated mice, irrespective of the day of harvest (day 3 or day 18) or the route of administration (i.t. or systemic), presented high polymorphonuclear infiltration.

To evaluate immune infiltration, tumors were disaggregated and digested to prepare cells in suspension, and cells were incubated with CD45+ and CD8+ antibodies and analyzed by flow cytometry. The amount of CD45+ T cells detected was similar in all groups, including the PBS-treated groups. However, we detected a higher level of tumor-infiltrating CD8+ T cells in the virus-treated groups. This high level of CD8+ T cells was also detected in the non-injected contralateral tumors of the ICOVIR17K-iRGD i.t. treated mice (Figure 4D). This observation suggested the viral activation of tumor-specific CD8+ T cells.

Taking advantage of certain immunogenic neoepitopes derived from mutations present in CMT64,^11^ we then assessed the tumor-specific T cell response in splenocytes by interferon-gamma (IFN-γ)-Enzyme-Linked ImmunoSpot (ELISpot). The exome of our subclone CMT64.6 was sequenced to check whether it carries the same mutations as the parental model CMT64. Of the seven main immunogenic mutations detected for CMT64 upon oncolysis with adenovirus,^11^ three were present in the CMT64.6 subclone (Rab13, Ndufs1, and Arhgef10). We therefore checked whether treatment with ICOVIR17K-iRGD induced immune responses to these neoepitopes. The three neoepitopes were synthesized as minimal binding-motif peptides and incubated with splenocytes in an IFN-γ-ELISpot assay. The peptide E1B192 and the adenovirus hexon peptide pool PepTivator were used to detect anti-viral responses. Infected and non-infected CMT64.6 cells, as live cells or cell lysates, were used to further discriminate between anti-tumor and anti-viral immune responses. An anti-tumor immune response was found against the Nduf1 mutation in one mouse (out of six) treated with the virus i.t., suggesting that i.t. administration was better than i.v. administration at inducing tumor-specific immune responses. Otherwise, the immune response was mainly directed against viral antigens (E1B199, PepTivator, and infected cells) in mice that had been treated with viruses, independent of the administration route, pointing at the strong immunodominance of the virus compared to the tumor neoepitopes (Figures 4E and 4F).

**Figure 5:**
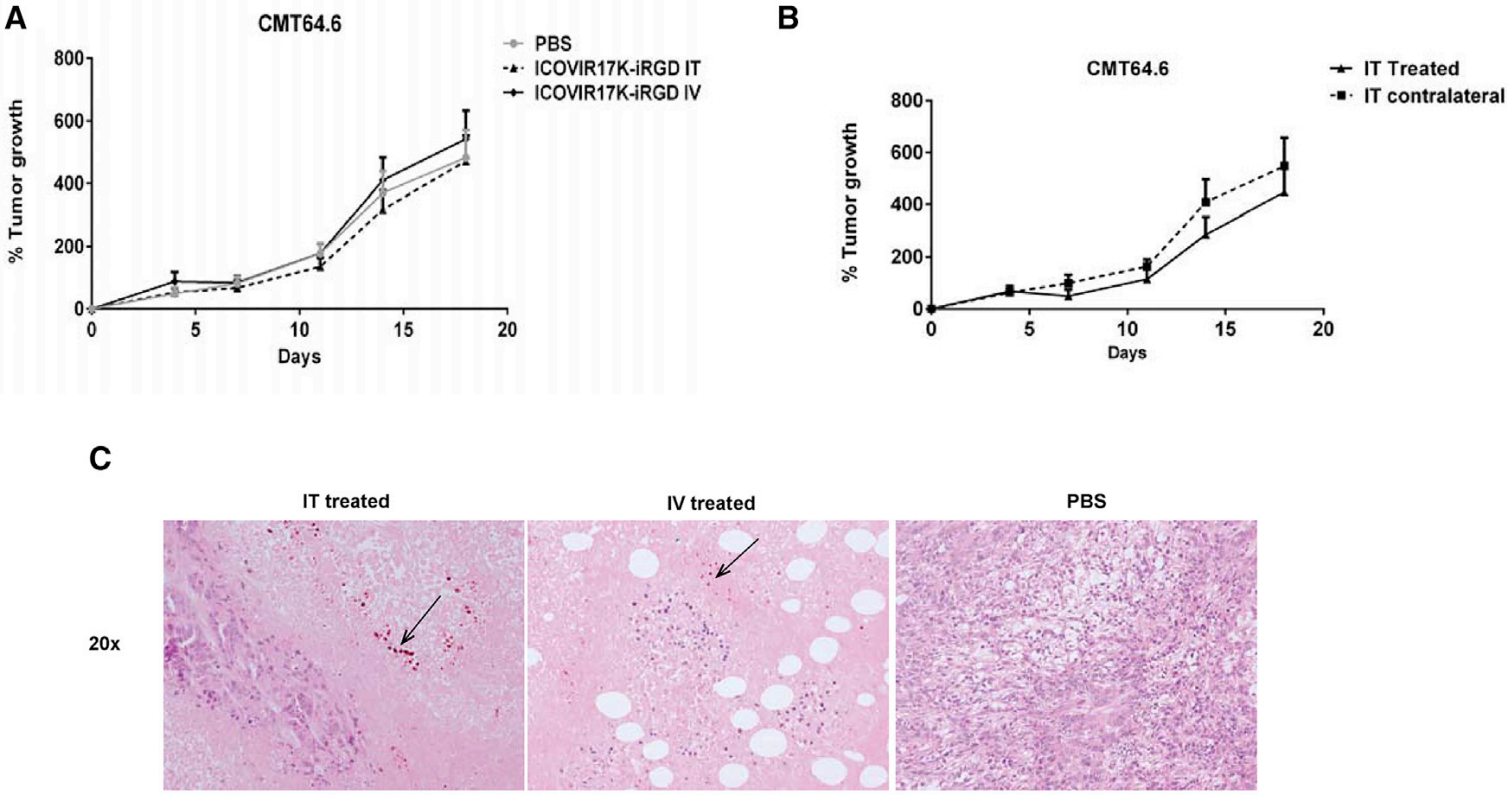
Anti-tumor Efficacy of Systemic or i.t. Administration of ICOVIR17K-iRGD in Immunodeficient Mice Athymic nude mice bearing CMT64.6 murine lung carcinoma subcutaneous tumors were treated via an i.v. or i.t. single dose of PBS or 3 × 10^10^/mouse of ICOVIR17K-iRGD. (A) Percentage of tumor growth at the indicated days is plotted (n = 11–12). (B) Comparison of injected and non-injected contralateral tumors of the i.t. mice group. Percentage of tumor growth (mean ± SEM) at the indicated days is plotted (n = 5–6). (C) Immunohistochemical staining of E1A of a representative tumor treated i.t. The arrow points to positive virus E1A staining.

To further determine the role of the immune system in the observed partial control of tumor growth, we performed a similar study to assess virus efficacy in immunocompromised mice. Although the mouse cell clone CMT64.6 is more permissive to Ad5 than the parental cell line CMT64, the levels of virus replication are still low compared to human cells. This limits the oncolytic effect mediated solely by replication and virus spread in the absence of an immune response. CMT64.6 subcutaneous tumors were grown in both flanks of athymic nude mice and treated with ICOVIR17K-iRGD via i.v. or i.t. administration routes. Tumors were measured twice per week and collected for immunohistochemistry at the end of the experiment. No treatment was effective despite the detection of virus i.t. (Figure 5). The amount of virus observed in tumors was low and localized to a few areas, indicating that replication was not efficient enough to affect tumor size. The lack of tumor control in nude mice indicated that the activity of ICOVIR17K-iRGD against the CMT64.6 graft in C57BL/6 mice was immune mediated.

In summary, the newly generated virus ICOVIR17K-iRGD showed remarkable efficacy when tested in immunodeficient mice bearing a fully permissive human tumor xenograft. In contrast, no efficacy was observed in immunodeficient mice bearing a mouse tumor isograft due to the limited replication of the virus. However, efficacy was partially rescued against this isograft in the presence of the immune system.

## Discussion

Safe and effective cancer viral therapy depends on several factors. Our group has previously published on strategies for tumor-targeting, tumor-selective replication, and i.t. spread of oncolytic adenoviruses. With regard to tumor targeting, a clear advantage was shown with the replacement of the adenovirus fiber shaft heparan sulfate proteoglycan-binding domain by RGD^12^ and the insertion of an iRGD peptide at the fiber C terminus of ICOVIR15K.^6^ For better i.t. spread, we armed the adenovirus with a soluble form of the human sperm hyaluronidase.^13^ A combination of viral modification strategies, such as the K mutation with the hyaluronidase expression in ICOVIR17K, confirmed the value of such procedures. Here, we combined the virus ICOVIR17K with the insertion of iRGD to generate the oncolytic virus ICOVIR17K-iRGD.

As shown in this study, the insertion of iRGD in a hyaluronidase-armed oncolytic adenovirus led to an increased efficacy in immunodeficient mice. Taking into account that the infectivity and cytotoxicity enhancement associated with iRGD was not high (Figure 1), the striking anti-tumor activity of ICOVIR17K-iRGD compared to that of the parental ICOVIR17K observed *in vivo* may be explained by the increased extravasation and tumor penetration effect of iRGD, properties that are possible *in vivo* only. The virus was also able to show some efficacy in immunocompetent mice, although to a less apparent extent. However, the immunocompetent model is limited by poor replication of human adenovirus in mouse cells. Studies have been performed in murine models to evaluate their permissiveness to human adenovirus.^8^, ^14^ We used the model clone CMT64.6 *in vivo*, which is more permissive than its parental model CMT64,^12^ though we were not able to reduce total tumor mass or detect viral protein by immunofluorescent staining *ex vivo*. Nonetheless, in immunocompetent mice, we observed high polymorphonuclear infiltration that could contribute to anti-tumor efficacy and/or to virus clearance. To assess whether the anti-tumor efficacy in the immunocompetent model could be increased, we attempted i.t. administration. Despite clinical trials and pre-clinical studies of oncolytic viruses, so far there are no data about the best route of administration. Systemic administration is a more accessible route than the i.t. one; however, toxicity, tumor selectivity, and insufficient tumor delivery are caveats identified with the use of this route. Using ICOVIR17K-iRGD in immunocompetent mice, no differences were observed among the two routes of treatment regarding tumor size reduction and immune infiltration. Using immunohistochemistry, we were not able to detect virus at the late stage of the experiment for any of the treatment routes, but it was detectable at the early stage for tumors treated i.t. The systemic route had the same efficacy as the i.t. route, though the virus was not even detected at the early time points by the systemic route. We speculate that upon i.t. administration, the amount of virus is higher near the injection needle track, which favors its detection at early time points. In contrast, i.v. administration delivers the virus at a lower concentration, which may be insufficient to be detected by immunohistochemistry in this semi-permissive model, but the virus may be more evenly distributed throughout the tumor. Regardless of these possible distribution patterns, it seems that the limited presence of virus during the initial few days post-administration is sufficient to affect tumor size long term.

Studies suggest that oncolytic adenoviruses can trigger a CD8+ T cell-mediated anti-tumoral response.^8^, ^11^ Hence, we tried to determine whether there was a relationship between efficacy and immune infiltration. Among the seven virotherapy-responsive neoepitopes reported for the cell line CMT64,^11^ our clone CMT64.6 shares only three. We detected a response toward one of these neoepitopes (Ndufs1) in one mouse of the i.t. treated group, but the anti-tumor efficacy was not better for this mouse compared to others of the same group. Even though the observed response toward the epitope was only found in one animal treated i.t., we observed an increase in CD8+ T cells in tumors as a consequence of oncolytic virus administration. A larger number of candidate neoepitopes should be explored to better evaluate the immune response induced by the oncolytic adenovirus. We hypothesize that the immune system generates both anti-viral and anti-tumor adaptive immunity, though the anti-viral response is dominant. Despite being mostly anti-viral, we believe the immune response contributes to tumor reduction, because we did not observe efficacy in immunocompromised mice with same murine model CMT64.6 (Figure 5). A similar finding was observed in a previous study with the colorectal carcinoma cell line CMT93.^14^ The combination of oncolytic viruses and checkpoint inhibitors is a hot area of preclinical and clinical research.^15^ This combination has been shown to increase the immune response to neoepitopes in the CMT64 model and to overcome the resistance to programmed cell death protein 1 (PD1) immunotherapy.^11^ It will be of interest to see whether genetic modifications related to oncolytic potency can also increase anti-neoepitope responses.

One study demonstrated that mesenchymal stem cells (MSCs) infected with oncolytic adenoviruses increase tumor-infiltrating lymphocytes,^8^ We believe that such a combination not only provides delivery protection to the virus but also increases anti-tumoral immune responses. In addition, full virus permissiveness or replication in the animal model could improve the prospects of eliciting anti-tumor immunity. A study of a murine glioma model suggested that the cell death caused by virus replication is required to induce a significant anti-tumor immune response.^16^ Accordingly, the virus ICOVIR17K-iRGD might be more effective in clinical studies in which the replication will be more efficient than in the mouse model. Finally, immunovirotherapy may confer benefit to other strategies aimed at inducing anti-tumor immune responses, such as CD40L costimulation,^17^ bispecific T cell engagers (BiTEs),^18^ incorporation of immunostimulatory genes such as interleukin-12 (IL-12),^19^ or checkpoint inhibitors.^20^

In summary, ICOVIR17K-iRGD was effective against human A549 tumors in immunodeficient mice. In immunocompetent mice with syngeneic tumors, partial tumor control mediated by the immune system was observed despite a strong anti-viral response and low replication levels. This model opens the possibility of additional studies on the use of transgenes or combinations with other therapeutic or immunostimulatory agents to improve immunovirotherapy with oncolytic adenoviruses.

## Materials and Methods

### Cell Lines

Human lung adenocarcinoma A549 and murine lung carcinoma CMT64.6 were cultured in DMEM (PAA, Les Mureaux, France) supplemented with 10% fetal bovine serum (Gibco, Carlsbad, CA, USA) and penicillin-streptomycin (Invitrogen, Carlsbad, CA, USA). The A549 cell line was obtained from the American Type Culture Collection (ATCC, Manassas, VA, USA). The CMT64.6 cell line was described previously.^8^ All cells were routinely tested for mycoplasma.

### Generation of Recombinant Adenovirus

All genetic modifications were performed following a recombining protocol adapted from Stanton et al.^21^ based on homologous recombination in bacteria and a positive-negative selection method with the RpsL-Neo selection cassette. To generate pAdZ-ICOVIR17K-iRGD, a fragment containing the (GGGGS)3 linker, the iRGD peptide (CRGDKGPDC), a stop codon, and a polyadenylation site was inserted, substituting for the fiber stop codon in the pAdZ-ICOVIR17K plasmid (ICOVIR17K) carrying all genetic modifications of ICOVIR17K. The fragment was inserted in the C terminus of the plasmid pAdZ-ICOVIR17K, generating the new plasmid pAdZ-ICOVIR17K-iRGD. This plasmid was transfected by the calcium phosphate standard protocol into HEK293 cells to generate ICOVIR17K-iRGD. The virus was plaque purified in A549 cells and further amplified and purified following a standard cesium chloride double-gradient protocol. Both plasmid and virus were sequenced to confirm that they had all indicated modifications.

The generation of adenoviral vectors AdGLK and AdGLK-iRGD containing the GFP-luciferase fusion protein under the control of a cytomegalovirus promoter substituting for the adenovirus E1 region has been described previously.^6^

### *In Vitro* Infectivity and Cytotoxicity Assays

The cell lines A549 and CMT64.6 were plated at 1 × 10^5^/well in 96-well plates, and infected with different MOIs with the indicated adenoviral vectors. After 24 hr, cells were trypsinized, and GFP-expressing cells were quantified by flow cytometry using the Gallios cytometer (Beckman Coulter, Indianapolis, IN). Cytotoxicity in A549 cells was performed as described previously.^6^ At day 6 post-infection, the total amount of protein content was quantified by bicinchoninic acid (Pierce Biotechnology, Rockford, IL, USA), and the IC_50_ value was determined from the dose-response curve by standard non-linear regression (GraphPad Prism 5; GraphPad, La Jolla, CA).

### Evaluation of Anti-tumor Efficacy *In Vivo*

*In vivo* studies were performed at the Institut Català d'Oncologia- Institut de Investigació Biomèdica de Bellvitge (ICO-IDIBELL) facility (Barcelona, Spain), AAALAC unit 1155, and approved by IDIBELL’s Ethical Committee for Animal Experimentation. Lung adenocarcinoma xenograft tumors were established by implanting 5 × 10^6^ A549 cells subcutaneously into both flanks of 7-week-old female athymic nude/nude (nu/nu) mice, When A549 tumors reached 200 mm^3^, mice were randomized, distributed into three groups (n = 12 tumors/group), and treated systemically with a single dose of 200 μL of PBS or 4 × 10^10^ vp/mice of ICOVIR17K or ICOVIR17K-iRGD, injected through the tail vein. PBS was used as a vehicle.

Efficacy in the immunocompetent model was established by implanting of 2 × 10^6^ CMT64.6 into 8-week-old C57BL/6 flanks. When tumors reached 100 mm^3^, mice were randomized, distributed into groups (n = 12 tumors/group), and treated with PBS or 3 × 10^10^ ICOVIR17K-iRGD. PBS was used as a vehicle.

In efficacy experiments, mice were monitored twice a week. Tumor volume was measured with a digital caliper and defined by the equation V (in cubic millimeters) = π/6 × W^2^ × L, where W and L are the width and the length of the tumor, respectively. The two-tailed Student’s t test was used to study the statistical significance difference in the tumor growth between the treated groups.

### ELISpot

ELISpot assays were performed to determine IFN-γ release by activated splenocytes using a 96-well filtration plate with an Immobilon-P membrane (MultiScreen HTS, Merck, Kenilworth, NJ, USA). Plates were activated with 35% ethanol for 5 min, washed five times with sterile water, incubated overnight with rat anti-mouse IFN-γ (BD AN-18, 4 μg/mL), and blocked with RPMI medium containing 10% fetal calf serum (FCS) and penicillin-streptomycin for at least 2 hr. A single-cell suspension of 2.5 × 10^5^ splenocytes/well was plated in 100 μL of RPMI medium including 2.5 × 10^5^ tumor cells or 20 μL of tumor lysate or 200 ng of peptide. After incubating overnight at 37°C and 5% CO2, plates were washed and stained with biotinylated anti-mouse IFN-γ (BD R4-6A2, 1 μg/mL) and incubated for 2 hr, followed by streptavidin conjugate enzyme (E236, Sigma) and appropriate washing. After drying, spots were counted using the ELISpot Reader (AID, Strasberg, Germany).

The peptides H2-Q2 (TWQLNGEEL), Rab13 (SDKKNNKCL), Ndufs1 (AAVSNMVQKI), Ppat (DPYGNRPLCM), Gsta2 (LHHFNARGRM), chd2 (APLQNSLKEL), and Arhgef10 (AWIENPEEAI) were synthesized by Biomedal (Seville, Spain), and the sequence of the peptides was as reported.^11^ The peptide E1B192 (VNIRNCCY)^22^ was synthesized by GenScript USA (Piscataway, NJ, USA). The hexon peptide pool PepTivator AdV5 Hexon was purchased from Miltenyi Biotec (Auburn, CA, USA). For all peptides, purification was >75%, and they were dissolved in DMSO. The final concentration of each peptide used for stimulation was 2 μg/mL.

### Tumor-Infiltrating Lymphocytes

Tumors were mechanically disaggregated and incubated for 30 min at 37°C in RPMI supplemented with 0.25 mg/mL collagenase I (Thermo Fisher Scientific, Waltham MA) and 0.1 mg/mL DNase (Roche, Basel, Switzerland). Single-cell suspensions were prepared by passing the digested tumors through a 70-μm cell strainer. Cells were stained for viability with Live/Dead fixable stain (Thermo Fisher Scientific), followed by incubation with fluorescein isothiocyanate (FITC)-CD45 (30-F11) and antigen presenting cells (APCs)-CD8a (53-6.7), both from BioLegend (San Diego, CA). Flow cytometry analysis was performed with a Gallios cytometer (Beckman Coulter).

### Histology and Immunohistochemistry

For immunohistochemistry in paraffin-embedded tissue, mice were sacrificed and tumors were fixed with paraformaldehyde overnight, and then tumors were preserved in paraffin. Sections of a 4-mm thickness were cut from paraffin blocks, followed by the standard protocol. Primary antibody incubation was performed using an anti-Ad2/5 E1A antibody (SC-430, Santa Cruz, Dallas, TX, USA) diluted 1/200 in PBS, and then slides were stained with H&E.

### Statistical Analyses

Differences among groups were estimated with the two-tailed Student’s t test or ANOVA with GraphPad Prism 5 (GraphPad, La Jolla, CA, USA). Differences were considered statistically significant when p < 0.05.

## Author Contributions

A.A.A.-Z. and R.A. designed the experiments, analyzed data, and wrote the manuscript. A.A.A.-Z. performed the experiments. R.M., C.A.F., M.A.-B., M.F., and J.S. contributed experimentally to *in vitro* and *ex vivo* experiments. L.A.R. provided expertise and feedback. All authors reviewed the manuscript.

## Conflicts of Interest

No potential conflicts of interest are disclosed.
